# Loperamide-Induced Acute Pancreatitis

**DOI:** 10.1155/2013/517414

**Published:** 2013-10-02

**Authors:** Halla Vidarsdottir, Hanna Vidarsdottir, Pall Helgi Moller, Einar Stefan Bjornsson

**Affiliations:** ^1^Department of Surgery, Helsingborg Hospital, 25187 Helsingborg, Sweden; ^2^Department of Anaesthesiology and Intensive Care, Landspitali University Hospital, 101 Reykjavik, Iceland; ^3^Department of Surgery, Landspitali University Hospital, 101 Reykjavik, Iceland; ^4^Faculty of Medicine, University of Iceland, 101 Reykjavik, Iceland; ^5^Department of Gastroenterology, Landspitali University Hospital, 101 Reykjavik, Iceland

## Abstract

Acute pancreatitis is a common disease leading to hospitalizations, most often caused by gallstones or alcohol. We present a case of a patient diagnosed with acute pancreatitis considered to be due to loperamide treatment for diarrhea.

## 1. Case Report

**Figure 1:**
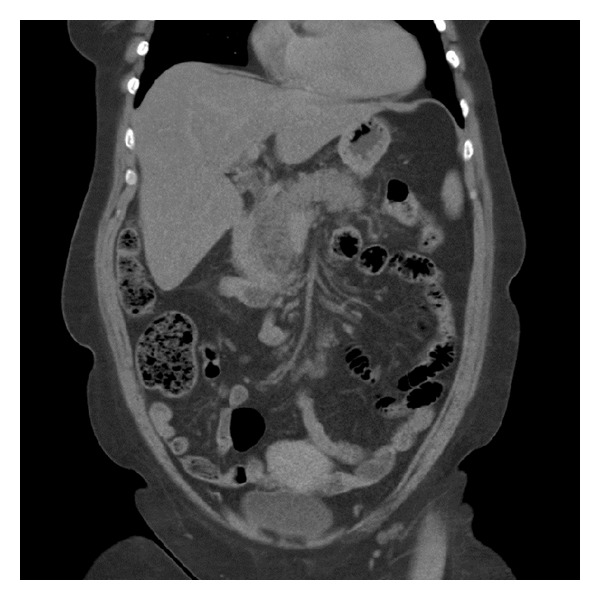
A computed tomography scan at the day of admission showing pancreatitis changes.

A 58-year-old woman with a history of hypertension, hypothyroidism, and cholecystectomy presented to the emergency department with a 7-day history of abdominal pain, nausea, and vomiting. The pain started after she began to take loperamide for diarrhea. She started having diarrhea 10 days before admission and took loperamide for two days, 10 mg on the first day and then 6 mg on the second day. Her daily medications were thyroxin, hydrochlorothiazide, and atenolol. She did not drink alcohol or smoke. At initial clinical examination, she had normal vital parameters (blood pressure, pulse, oxygen saturation, respiration rate, and temperature) and palpation tenderness in epigastrium. She had elevated lipase 2513 U/L (normal < 300 U/L) and amylase 124 U/L (normal < 120 U/L). WBC was 12,5 mmol/L and CRP was 63 mg/L. Hemoglobin, electrolytes, arterial blood gas, creatinine, urea, and liver functions tests including bilirubin were within normal limits. Abdominal CT scan showed the status after cholecystectomy and acute pancreatitis (Figure 1). MRCP showed the status after cholecystectomy, diameter of the common bile duct was 5 mm, and there were no signs of gallstones. Her calcium level was 2,22 mmol/L.

Loperamide was discontinued, her condition improved rapidly, and she was discharged three days after her admission. The patient has not suffered further attacks of pancreatitis and is doing well approximately 12 months after her hospitalization.

## 2. Discussion

Acute pancreatitis is a common disease leading to hospitalization, with incidence figures ranging from 14.6 to 32 per 100.000 [1, 2]. In most series, the vast majority of patients have gallstones- or alcohol-induced pancreatitis. Drug-induced pancreatitis is relatively rare [3]; however, 525 different drugs are listed in the World Health Organization (WHO) database suspected to cause acute pancreatitis as a side effect. Many of them are widely used to treat highly prevalent diseases. The true incidence is not entirely clear since only few systematic population-based studies exist [4]. In a recent cohort study, 3,4% of the patients had drug-induced pancreatitis [3]. Some drugs have pancreatitis documented as a side effect such as azathioprine [5], and opiates have also been shown to lead to pancreatitis in most series investigating this [3–5].

Loperamide, a peripheral acting opiate [6], has very rarely been associated with pancreatitis. The first case report on this possible association, published in French, described a 57-year-old woman with a history of a cholecystectomy, who had taken 4 mg of loperamide therapeutically and presented 2 hours later with lower abdominal pain and an elevated amylase that normalized in several days. Her abdominal pain resolved spontaneously within several hours [7]. From this first report, pancreatitis has been reported in 2 cases to be due to loperamide overdose [8, 9]. An 18-year-old woman had taken an overdose of loperamide and presented an hour later with elevated lipase and amylase, which normalized within 24 hours. Abdominal ultrasound was normal [8]. Lee et al. reported a previously healthy 17-year-old woman who had taken an overdose of loperamide and presented 2 hours later with mild abdominal pain and elevated lipase and amylase. Abdominal ultrasound and computed tomography showed a normal pancreas. She recovered spontaneously [9]. All the previously reported cases have revealed normal pancreas on ultrasound or abdominal CT scan. However, in the current case, the abdominal CT scan showed clearly inflamed pancreas (Figure 1). Our patient came to the hospital one week after she became symptomatic, but in the other published cases the patient presented after few hours.

Loperamide is a synthetic agonist of peripheral opiate receptors and used in treatment of diarrhea [6]. The effect on diarrhea is primarily due to an inhibition of intestinal secretion and gut motility [10]. The mechanism of pancreatic injury is thought to be by two different mechanisms. A study on 8 healthy people showed that intraduodenal output of amylase decreases after loperamide ingestion [6]. Being an opiate it probably causes a spasm at the sphincter of Oddi in a similar waytomorphine. A study on 6 healthy people has shown that loperamide causes dose-dependent inhibition of pancreatic polypeptide release mediated by vagal-cholinergic pathways [10]. The pancreatic polypeptide suppresses the secretion from exocrine pancreas, and it has been proposed that it most likely increases exocrine pancreas secretion [9].

In summary, the current case presented is the first one published in English on loperamide associated pancreatitis not associated with overdose. Thus, it seems likely that loperamide in therapeutic doses can lead to clinically important pancreatitis with inflammatory changes seen in the pancreas on imaging studies.
